# Analysis of Fatigue and Residual Strength Estimation of Polymer Matrix Composites Using the Theory of the Markov Chain Method

**DOI:** 10.3390/ma18143229

**Published:** 2025-07-08

**Authors:** Rafał Chatys, Mariusz Kłonica, Ilmars Blumbergs

**Affiliations:** 1 Faculty of Mechatronics and Machine Design, Kielce University of Technology, al. 1000-lecia P.P.7, 25-314 Kielce, Poland; chatys@tu.kielce.pl; 2 Department of Production Engineering, Faculty of Mechanical Engineering, Lublin University of Technology, Nadbystrzycka 36 Street, 20-618 Lublin, Poland; 3 Faculty of Engineering and Management, Riga Aeronautical Institute, 9 Mezkalna Street, LV-1058 Riga, Latvia; i.blumbergs@rai.lv

**Keywords:** component of composite, destruction, fatigue strength, residual strength, curing process, Markov theory

## Abstract

This paper deals with an important issue, which is the influence of failure caused by the quality of matrix post-curing on the strength of complex and difficult materials of the “new generation” such as fibre composites, particularly with a polymer matrix. In recent years, significant advances in the field of adhesive materials chemistry have led to the constant development of bonding technology. The effectiveness of bonding depends, to a large extent, on the suitable selection of the adhesive and the use of appropriate surface treatment technology. It is difficult to imagine virtually any modern industry without adhesive joints, be it the aircraft, aerospace or automotive industries, which simultaneously highlights the great importance of adhesives and adhesive materials for the present-day economy. In modern technology, it is extremely important to obtain the right combination of modern construction materials. The statistical analysis of the components showed the complexity of the layered composite structure. The proposed model of the weakest micro-volume developed in this study indirectly reflects the experimentally based curing variables that affect the stresses of the components in the composite (laminate) structure. The strength of fibrous composite structures based on the Markov chain theory considers technological aspects during hardening. The model proposed in the paper was validated on the basis of examples from the literature and experimental data obtained in the research project. The numerical results are in good agreement with the literature database and measurement data. The presented model could be a novel method, which allows better insight into the curing process of epoxy resins.

## 1. Introduction

The recent use of a large range of polymer-matrix components forces us to develop new models for estimating the strength properties of laminates (fibre composites) or combinations thereof, including the development of procedures for the moulding and manufacture of resin systems.

Epoxy resins (as chemically curable compounds), which are able to crosslink at elevated temperatures when cured with aromatic diamines (or phthalic or maleic anhydride), are associated with a running chemical crosslinking reaction, i.e., the formation of crosslinks between chains. The crosslinking reaction, which is polyaddition (or ionic polymerisation), proceeds without volatile by-products (low-molecular-weight precipitates), based on bisphenol A glycidyl ether (DGBEA diglycidyl ether of bisphenol A) [1,2,3]. 

It should be noted that additives (such as thixotropic compounds) added to the resin system reduce viscosity during shear (flow) despite maintaining a constant temperature and shear rate. This means that when shear stresses on the polymer matrix system cease (e.g., cessation of mixing, flow, etc.), its viscosity increases spontaneously. This is of great practical importance, especially with the formation of a gel structure. As a result of the formation of weak hydrogen bonds between the grains of thixotropy promoters (compounds containing at least two groups capable of forming a hydrogen bond with additive grains) or polymer molecules fulfilling this role.

The hydrogen bonds are relatively weak, and the shear forces inducing the flow destroy these bonds, leading to reduced interactions between the particles of the thixotropic additive. As a result of the cessation of shear, the gel structure is rebuilt and the viscosity of the system is increased. Depending on the type of polymer and additives used, as well as the shear rate, these processes run faster or slower, and the time for the restoration of the thixotropic structure or its destruction can be shorter or longer.

This is why the curing process, which determines the quality of the composite, is so important. Hence, non-isothermal curing as a result of non-compliance with reaction parameters can result in incomplete curing or the entrapment of volatiles or the formation of voids, leading to matrix degradation. Non-isothermal measurement curves determine the nature of the curing reaction (i.e., heat flow as a function of temperature via DSC scanning calorimetry for DGEBA), as a result of a multi-step reaction (due to the occurrence of gelation and vitrification [4,5]).

It needs to be stressed that the process of destruction as a result of the increase in internal temperature caused by the exothermic chemical reaction of the epoxy (i.e., the exceeding of the so-called “resin system life” [6]) is influenced by the quality of the polymer matrix composite (i.e., the amount of trapped volatiles or voids) and, consequently, the normal and tangential interlayer stresses (τ), which visually cause the free edge of the laminate to “swell” [7,8]. White and Hahn [9] and Ciriscioli et al. [10], respectively, proposed the optimal temperature cycle and algorithm [11], which may reduce the residual stresses and voids inside the composite structure (laminate).

The influence of cyclic thermal loading on the strength, both static and dynamic, of lap joints is relatively poorly known, especially the aspect of the mechanical and chemical changes occurring within the adhesive. For the most reliable prediction of the adhesive joint performance of modern construction material substrates under variable thermomechanical loading conditions, it is necessary to understand the behaviour of the adhesive subjected to shock changes in the operating temperature. Thermal fatigue of cured adhesives may imply aging processes and thus a change in the mechanical properties.

The non-uniform temperature distribution over the curing cycles (i.e., the time of the temperature exothermic peak) affects the residual deformation, and the shear and fracture properties of the interlayer composite for glass–epoxy laminates or static and fatigue tests (S-N curves), respectively, are presented in the work for a laminate for wind turbine blades with the thickness of 84 ply (55-60 mm [12]) and the production of thick laminates [13]. On the other hand, the flow and flow rate of the polymer matrix resin system during curing for different pressures shows good agreement with the Springer–Loos model [14] for thin rods or a layer of porous plates. 

Therefore, two important issues for laminate quality are addressed in the literature during modelling of the flow of a resin system:

- Precise prediction of the temperature history [15,16,17] that acts against a resin system turning into a gel before the mould is filled (or the resin system curing too fast within the composite [18,19,20]);
- Predicting and measuring the flow patency in order to calculate, with acceptable accuracy, the temperature as well as velocity of the resin system situated within the mould [21,22,23].

This information has important implications for designers and technologists, who have to decide whether to use laminates or adhesive joints as connections under thermomechanical loads (thermal shocks) other than plasma coatings [24,25]. The fatigue process of adhesive joints in particular is the result of the interaction of stresses cyclically varying in time. This process causes a reduction in the strength and durability of the joints and consequently leads to the failure of the structure at a maximum stress value lower than the static strength [26]. The problem of thermal fatigue of adhesive joints and the effects of this fatigue on long-term and failure-free operation is still being analysed [27]. Failure to comply with technological parameters, including the degree of postcure, leads to a bigger chance of delamination at the edges of the samples (mostly within the layers [28]). An understanding of this phenomenon in laminates becomes apparent when analysing the arrangement of layers in the composite (and thus taking into account the distribution of stress within the structure [29]).

An option when describing damage accumulation (damage accumulation) via the simple statistical or fatigue properties of polymer matrix composite components is Markov networks [29], which is not a novelty [30,31]. 

Random variables or vectors describe the static phenomena, while stochastic processes describe phenomena which change in time as a “chain” [32,33,34] (the discrete time parameter case (as opposed to the continuous one), which the term “process” is associated with). The fatigue process is presented in more detail in [34,35,36,37] in many stochastic models.

Therefore, the problem of estimating the fatigue and residual properties of laminates (reinforced with glass fabric (UDO^®^ type E) with the structure [0/45/0]) or their combinations depends on the curing level of the resin system.

Previous studies have focused mainly on the study of the mechanical properties of composites [38,39], prediction of the strength of composite materials [6,26] or the study of adhesive joints [29]. Meanwhile, studies on the prediction of fatigue strength and its mechanism of composite adhesive joints subjected to variable thermal loads, which are extremely important in the aviation and automotive industries, are not fully understood. Therefore, in this article, the modelling of mechanical properties (e.g., E, σᵧ) from the degree of hardening was designed, taking into account the Markov chain theory, which provides a good correlation between the experimental results and the predicted ones. This paper describes a model for determining the strength of a fibre composite based on the critical micro-volume, taking into account the failure stresses of single-layer epoxy joints after thermal shocks relative to the samples before thermal shocks. The use of the Markov model allowed for probabilistic analysis of material degradation under assumed cyclic loading conditions, as well as an analysis of the influence of parameter uncertainty on the predicted residual life.

## 2. Materials and Methods

The adhesive composition used in the study consisted of epoxy resin (LH-160) (Czech republic, Havel Composites) together with the addition of hardener (H-147) (Czech republic, Havel Composites) at an amount of 25%. With this material, the mechanical properties of the samples were measured (with DIN-EN ISO 527-2Stab 1B [40]). Some specimens were only seasoned at ambient temperature by subjecting them to 500 cycles of varying temperature in a thermal shock chamber. The thermal resistance of the cut specimens was increased by additional heat treatment at 86 °C, the so-called reheating, in order to get rid of the effect of large temperature values (Table 1) in the thermal shock chamber (which was used to determine the Young’s modulus value). The conditioning time of the samples in each chamber was 15 min, not including the time required for temperature stabilisation of the chamber. Table 1 summarises the preparation technologies for specimens bonded as single-layer joints.

**Figure 1:**
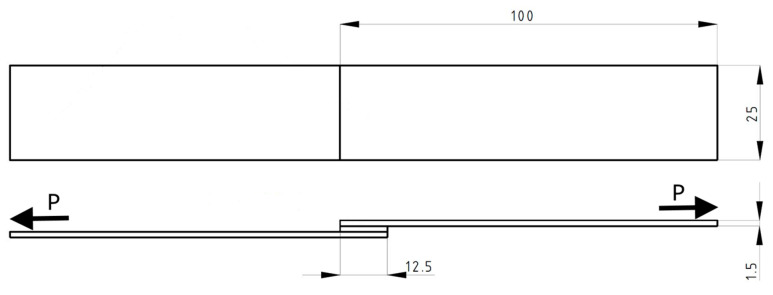
Schematic of a singly applied adhesive composition joint.

The adhesive composition used in the study was cured at an ambient temperature of 20–22 °C, with a relative humidity of (45–55)%. The unit pressure exerted on the surface of the samples used (Figure 1) during the bonding process was 0.2 MPa, and the curing time of the adhesive composition was set at 120 h.

Some of the specimens made as a single-layer adhesive joint were subjected to cyclic thermal loading.

Technological processes when forming composites (joints) in closed moulds, through the introduction of the resin system, rapid cooling or different thermal expansion of the matrix and reinforcement (fibre), will slow down the curing process of the material used [40]. This will result in the appearance of additional inherent stresses in the joint and, consequently, microdefects in the structure.

An assessment of these phenomena can be a measurement of the value of the dissipated energy occurring during a full loading and unloading cycle, or the energy associated with the adhesion forces between components. The change in activation energy during the destruction process will be a criterion for the subsequent rupture of the main adhesion bonds in the micro-volumes of the most stressed one. After the first cycles, the load stabilises as a result of self-stressing [41,42]. This is despite the fact that the fatigue properties of the polymer composition bond, combined with the low density and good wettability (of glass fibre with polymer), give a strong polymer–glass interfacial bond [40]. Also not to be forgotten is the ability to transmit loads at the boundary between these two phases. Giving the products the desired shape, transferring loads between fibres and shaping chemical and thermal properties are just some of the tasks and functions of the matrix. The correct choice of these parameters is a valuable result of the description and analysis of the failure processes occurring in polymer composites.

Temperature has a significant effect on the strength of single-layer adhesive joints, especially of epoxy systems. Changes of the order of a few or tens of degrees alter the properties of polymer matrix components. 

Varying heat loads affect the adhesive bond by introducing thermal stresses into the system, as well as changing the mechanical properties [7]. The exothermic (temper) peak, taking into account the time dependence of temperature, for the epoxy system used was estimated to be approximately 29 min.

Quite an important parameter is the determined glass transition temperature during the curing process for a system consisting of epoxy resin (LH-160), together with the addition of hardener (H-147) at 25 percent, which, under the assumed conditions, starts at 60°C (sample P2). 

The temperature of the sample (P1) was increased by 10 °C per min with a defined heat release as an exothermic process. The crosslinked sample was cooled and heated once more. The glass transition temperature (from three samples) of the cured epoxy system was estimated to be 76 °C and 86 °C, respectively, at the first and second heating. This means that the crosslinked sample was re-crosslinked during the first heating.

According to the tests carried out, Sample P1 was under-cured, while the other three samples were under-cured (P2–P3). Obviously, the glass transition temperature value from the second heating is larger in comparison to that from the initial heating, and this is due to the fact that the sample always under-cures after the first heating.

## 3. Experimental Research Results 

According to the tests of the adhesive composition with the resin system used (LH-160 and hardener H-147: 25%), an increase in the value of Young’s modulus was found after reheating at 80 °C for 2 h in relation to the samples before reheating. The largest increase of 36% was observed for samples prepared using P4 technology compared with samples prepared using P1 technology. In contrast, for samples before heat shock, the increase was 29% for samples prepared using P3 technology compared with samples prepared using P1 technology. A significant rise in scatter around the average value has also been observed for samples after reheating (variants P3 and P4) compared with samples before reheating (variants P1 and P2). The increase in the measure of scatter, which was the standard deviation, was fivefold.

Statistical analysis using the OSPPT criterion for a significance level of *α* = 0.05 confirmed the validity of the adopted distributions (normal and log-normal), as the OSPPt statistic falls within the Calfa hypothesis criterion (OSPPt < Calfa), which we cannot say about the E values for the elementary component bundles of the fabric with a measurement base of 250 and 450 mm (E_EPOXY_C2_ and E_EPOXY_C3_).

On the basis of the tests conducted, a 20% decrease in the failure stress of the single-layer epoxy (adhesive) joint after temperature shocks was observed in relation to the specimens before thermal shocks. On the other hand, the determined average static strength value (σ_stati_.) of the polymer composite (as a composition) consisting of elementary fibre bundles in the static tensile test was applied to determine the stress levels for fatigue testing, as well as the construction of the S-N curve.

The experimental analyses show that there is quite a large scatter in the strength properties of the composites. Therefore, the processing of the experimental results is mainly necessary when determining and testing the hypotheses adopted, the statistical distributions of the static strength (S_stati_) of the fibres of the bundles embedded in the resin and the specimens considering the different measurement base (Table 2).

**Figure 2:**
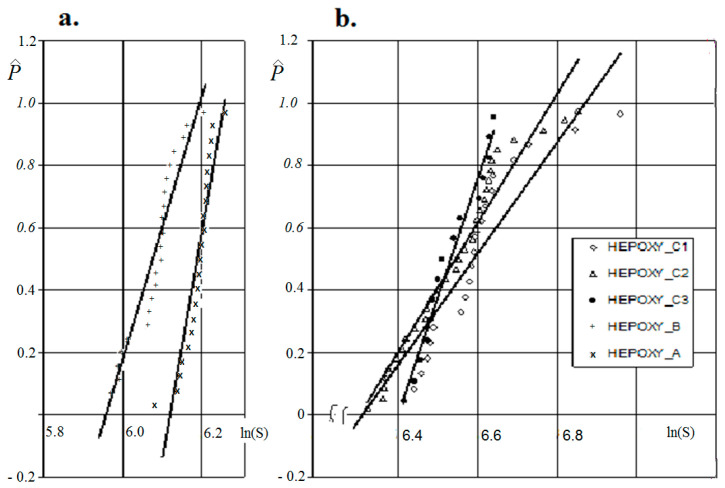
Tensile strength obtained from statistical analysis with different L_BP_ measurement bases [37], for composite (**a**) and elementary fibre bundles (**b**).

The graphical relationship ${\hat{P}}_{i}$–ln(*S*) shows that the strength of the elementary fibre bundles with different L_BP_ values is significantly higher than that of the composite samples (Figure 2). Considering the measurement base of the specimens, a higher average fibre bundle strength of approximately 30 and 15% should be mentioned for specimens with a measurement base of 250 and 120 mm, respectively.

The graphical representation of differences as an empirical density function ${\hat{P}}_{i}$–ln(*S*) allows the intensity and extent of changes in the strength of composites or fibre bundles to be assessed

(1) $$\hat{P}=(i-1/3)/(n+1/3)$$

where

i is the ordinal number of the expected value in the ordered set of samples;
n is the total set size.

With regard to the strength scatter plots for both the fibre bundles and the composite, it can be seen that the regression line for Specimen EPOXY_C3 differs from the nature of the regression lines for Specimens EPOXY_C1 and EPOXY_C2. The rationale for the differences above is the influence of scale effects and the composite forming technology (in our case, the specimens made).

It can also be seen that the strength ranges of the fibre bundle samples are different due to the fact that it is never possible to obtain two identical samples of composite and elementary fibre bundles, as each composite sample or sample as an elementary fibre bundle contains a different number of broken fibres that do not carry the load in the material.

It was also found that a small change in the length of the specimen significantly affects the strength value of the sample. This is due to the fact that a longer measuring base of the sample has more imperfections, which are the reason for its weakening. The validity of the proposed confidence intervals for the mean strength of composite samples with different L_BP_ values was verified using the Smirnov–Kolmogorov criterion (S-K) (Table 3), i.e., a distribution characterised by the maximum difference between the experimental F*(*x*) and theoretical *F*(*x*) data as a function of the distribution.

(2) $$D_{n}=\sup\left|F^{*}(x)-\left.F(x)\right|\right.=\max\left|D_{n}^{+},\left.D_{n}^{-}\right|\right.$$

where

$D_{n}^{+}=\underset{1<i<n}{\max}\left(\frac{i}{n}-F_{i}\right)$ is the upper limit;
$D_{n}^{-}=\underset{1<i<n}{\max}\left(F_{i}-\frac{i-1}{n}\right)$ is the lower limit;
*n_i_* is the number of meanings in the set;
*F*(*x*) is the distribution function.

If the resulting value of D* = $D^{*}=\left(D_{n}-\frac{0.2}{n}\right)\cdot \left(\sqrt{n}+0.26+\frac{0.5}{\sqrt{n}}\right)$ does not meet the conditions in this paper, then the distribution we assumed for n experimental database is proved to be wrong. Here, for composite samples with different measurement bases, the assumed condition is satisfied.

Due to the fact that some structures are subjected to high loads with a small number of cycles, it was decided to carry out the tests up to 500 cycles at a fixed stress level for the joints. An example of the S-N curve and residual strength for a specified stress level is shown in Table 4 and Table 5.

## 4. Modelling the Fatigue Strength of a Laminate, Taking Markov Chain Theory into Account

The model assumes the fatigue failure of a specimen made of n fibres in a matrix based on the estimation of a critical micro-volume not only in the ply but in n layers (laminate–ply composite).

**Figure 3:**
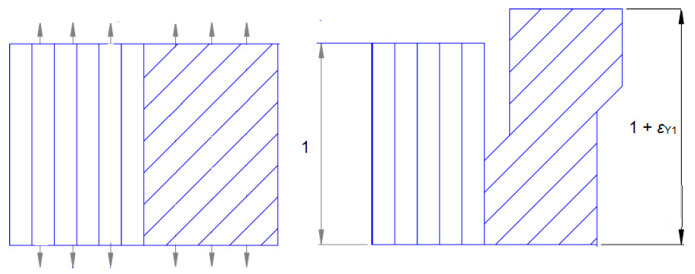
Model of the composite before and after load removal (description in text).

Elongated fibres or their bundles (working in the elastic range) operate together with a plastic matrix. The theory behind Markov chains [29] includes the operation of the matrix (Figure 3) and also the other layers of fibres operating along the load (at various angles of alignment within the elastic range). 

The angled volume 45° lines in Figure 1 symbolise the accumulation of irreversible plastic deformation. Before the deformation of the plastic part of the specimen reaches *ε*_Y_, the working fibres in the elastic range and the brittle matrix work together. Once the load is removed, internal stresses develop in the specimen: tension in the elastic and compression in the plastic part of the specimen. If the deformation exceeds *ε*_Y_, the composite (composed of longitudinal fibres or bundles of fibres working in the elastic range) is destroyed and, consequently, the whole specimen. This graphical representation of the material is symbolic. More figuratively, it describes metals, where the accumulation of plastic stresses is related to flow (for metals, displacement along slip planes). We will assume that each instance of flow, in the mathematical description, leads to corresponding changes in the Markov chains and, in the physical description, to the appearance of a permanent plastic deformation *ε*_Y1_.

### 4.1. Fatigue Model Assumptions

The model also assumes that the amount of elements r operating within the elastic range within a certain micro-volume falls by a certain value r_R_ (Table 6) with cyclic loading.

The destruction of the composite takes place after the accumulation of a critical number r_Y_, i.e., after the accumulation of critical plastic deformation, where r_Y_ and therefore *ε*_YC_ are the model parameters. Since the elements in the elastic and plastic working parts form a unity, subject to a common deformation, the accumulation of plastic deformation (irreversible deformation of the plastic working part) leads to the appearance of residual stresses (tensile stresses in the elastic part of the specimen and compressive stresses in the plastic part of the specimen).

The slow failure process of the specimen will be interpreted as a stationary Markov chain, whose states are defined by the number of destroyed, longitudinal elements and the number of yield stresses.

The state (i.e., destruction in the sample), as a stationary Markov chain process, determines the number of damaged elements along the axis (Case A) and the number of yield stresses of a certain value r_Y_ (Case B), and the set of z (r_Y_+1) blocks with (r_R_+1) internal states forming the matrix of the probability transformation. The input(i) and output (j) states’ indices were expressed as parts of the local indices i_Y_, i_R_, j_Y_ and j_R_.

The matrix of probability transitions (3), where all probability values situated below the diagonal equal 0, describes the fatigue strength [38]. The chosen probabilities may form conditional probabilities.

(3) $$P=\left[\;\begin{array}{ccccccc} q_{1} & p_{1} & 0 & & & \ldots & 0\\ 0 & q_{2} & p_{2} & 0 & & \ldots & 0\\ 0 & 0 & q_{3} & p_{3} & 0 & \ldots & 0\\ \ldots & \ldots & \ldots & \ldots & \ldots & \ldots & \ldots \\ 0 & & & \ldots & q_{r} & p_{r} & 0\\ 0 & & & \ldots & 0 & 0 & 1 \end{array}\right]\;\mathrm{where}:\;qᵢ=1-pᵢ,\;\;\;\;\;i=1,\;\ldots ,\;r$$

We can represent the probability transformation matrix as a set of (r_Y_ + 1) blocks with (r_R_ + 1) states within each block. We will introduce the indices i and j of the input and output states, expressed by the local indices i_Y_, i_R_, j_Y_ and j_R_, respectively with the formula:

(4) $$i=(r_{R}+1)(i_{Y}-1)+i_{R};\;j=(r_{R}+1)(j_{Y}-1)+j_{R},$$

In the example shown [(r_Y_ + 1)(r_R_ + 1) = 9] we have nine such states. The symbols p_R0_ and p_R1_ denote the probabilities of destruction of the corresponding number of elements (rigid) working in the elastic range (rigid—Case A), and p_Y0_ and p_Y1_ are the probabilities of the corresponding numbers of elements in which the yielding limit has been reached (yielding—Case B). We assume that the number of destroyed elements working in the elastic range after one step has a binomial distribution. If there are n_R_ still undestroyed elements left, we define the probability of destroying k_R_ additional elements by the formula

(5) $$p_{R}(i,j)=\left(\begin{array}{l} n_{R}\\ k_{R} \end{array}\right){\left(F_{R}\left(S_{R}\left(i_{R},i_{Y}\right)\right)\right)}^{k_{R}}{\left(1-F_{R}\left(S_{R}\left(i_{R},i_{Y}\right)\right)\right)}^{n_{R}-k_{R}}$$

where

$i=(r_{R}+1)(i_{Y}-1)+i_{R}$, $j=(r_{R}+1)(i_{Y}-1)+j_{R}$ $n_{R}=r_{R}-i_{R}$, $k_{R}=j_{R}-i_{R}$ at $0\leq k_{R}\leq n_{R}$, $1\leq n_{R}\leq (r_{R}-1)$;
$F_{R}(.)$ is the cumulative distribution function (cdf—random numbers) of the strength of the elements working in the elastic range and *S_R_* (*i_R_*, *i_Y_*) is the stress in the working part in the elastic range when the process is in a state of *i*.

The probability that, for the same process condition, the additional number of elements reaching yield stress (Case B) will be equal to k_Y_ can be described by the analogous formula

(6) $$p_{Y}(i,j)=\left(\begin{array}{l} n_{Y}\\ k_{Y} \end{array}\right){\left(F_{Y}\left(S_{Y}\left(i_{R},i_{Y}\right)\right)\right)}^{k_{Y}}{\left(1-F_{Y}\left(S_{Y}\left(i_{R},i_{Y}\right)\right)\right)}^{n_{Y}-k_{Y}}$$

where

$n_{Y}=r_{Y}-i_{Y}$, 1 $k_{Y}=j_{Y}-i_{Y}$ at $0\leq k_{Y}\leq n_{Y}$, $1\leq n_{Y}\leq (r_{Y}-1)$;
$F_{Y}(.)$ is the cdf distribution function of the relevant element numbers where the yield point was reached;
$r_{Y}$ is the critical number of elements in which the yield point has been reached;
$j_{Y}$ is the number of elements where the yield point has been reached;
(*j_Y_ −* 1) is the number identifying Case B;
$S_{Y}\left(i_{R},i_{Y}\right)$ is the stress in the plastic range, with a specified number of elements that have reached their yield point (*j_Y_ −* 1) and have been destroyed in the plastic range (*j_R_ −* 1).

A log-normal distribution was adopted for the points working in the plastic range

(7) $$F_{X}(x)=\Phi ((x-\theta_{0})/\theta_{1}),\;F_{Y}(y)=\Phi ((y-\theta_{0})/\theta_{1}),$$

where

*X*, *Y* are the strength limit of elements working in the elastic range and yield strength limit of elements working in the plastic range (on logarithmic scales);
$\theta_{0},\theta_{1}$ are the strength distribution parameters (mean and standard deviation);
Φ(.) is the function with a standard normal distribution.

We assumed a Markov chain where *r* defines irreversible states (as damage, whose accumulation leads to the destruction of the estimated critical micro-volume) and one absorbing state (in which the Markov chain reaches an absorbing state). The obtained probability values of the variable T (namely, the transformation’s inverse—Table 7) are described with the relation (10) and (11) using a cumulative distribution function (12).

### 4.2. Distribution of Fatigue Limits with a Limited Number of Cycles

Let us assume that the product of the matrix *P^i^* (15) and the vector b produces the column vector of the fatigue strength distribution, whose elements relate to the initial states of the Markov chain $\left(F^{\left(1\right)}\left(t\right)F^{\left(2\right)}\left(t\right),\ldots ,F^{\left(r\right)}\left(t\right)\right)$. Generally speaking, this could be applied to calculate the fatigue strength distribution function of a certain probability distribution in the initial states (if known, the fatigue strength distribution function has the form in the initial conditions p):

(16) $$F_{\sigma_{t}}\left(x\right)=\pi P^{t}b$$

The problem is to identify the relation between the probabilities p_i_ =1,...,r with the static strength distribution values of the composite elements and the fatigue loads. We assume that in the first step of the Markov chain (e.g., one or a thousand cycles), one element fails. If there are still (R-i) parallel components operating (possessing one and the same distribution function), with the static strength F(s), then the probability of the subsequent failure (of the remaining components) can be expressed as

(17) $$p_{i}=1-(1-F(s_{i}){)}^{\left(R-i\right)},$$

where

R is the initial number of elements, i is the number of damaged components and *s_i_* is the stress (loading) corresponding to a uniform distribution of load within the other (R-i) elements.

Thus, generally,

(18) $$s_{i}=\frac{SR=iS_{f}}{R-i}=\frac{S(1-iS_{f}/RS)}{1-i/R},\;$$

where

*S* is the initial (in the first step of the process) load in each element;
*S_f_* is the mean stress value which may still carry the load (at least at the start of the working composite components—the cumulative failure of the component that occurs in different sections).

The probability vector in the form of a Markov chain after loading (S_1_, n_1_), i.e., after n_1_ steps with stress S_1_, is defined as:

(19) $$\pi_{S_{1}n_{1}}=(1,0,\ldots )P_{1}^{n_{1}}$$

The residual strength σ_n1_ after loading (S_1_, n_1_), i.e., after n_1_ steps with stress S_1_, is measured only in unstressed specimens. The corresponding components of the probability distribution vector of unabsorbed (irreversible) Markov chain states are

(20) $$\pi_{S_{1}n_{1}}^{*}(k)=\pi_{S_{1}n_{1}}(k)/\sum_{m=1}^{m^{*}}\pi_{S_{1}n_{1}}(m),$$

where

$\pi_{S_{1}n_{1}}(k)$, *k* = 1,…, m^*^ are the vector components $\pi_{S_{1}n_{1}}$;
m^*^= (*r_Y_* + 1)(*r_R_* + 1) − (*r_Y_* + 1 + *r_R_*) are the total number of unabsorbed (irreversible) states.

### 4.3. Local Stress with the Estimated Fatigue Curve Equation and Residual Strength

The local stress in the model was obtained with the number of destroyed elements operating within the elastic range (e.g., Case A, or B), while the fatigue curve is determined by changing the numeration of states in which the composite is [37]. 

After destruction and the elements working in the elastic range, the new value of this cross-section will take the value from the relation (21), and the matrix of transformation of probabilities *P* (with *k*—degrees *P^k^*) has the form (27), taking into account the four conditions (Table 8).

(21) $$\left\{\begin{array}{c} S_{R}\cdot f_{R}+S_{Y}\cdot f_{Y}=S\cdot f,\\ \frac{S_{R}}{E_{R}}=\frac{S_{Y}}{E_{Y}}, \end{array}\right.$$

where

S is the average normal stress;
E is the modulus of elasticity, where the subscripts R and Y represent the elastic and plastic working parts, respectively.

If the lengths of the two sections are equal, then the equation is of the form:

(22) $$S_{R}=S\cdot f/(f_{R}+f_{Y}\cdot E_{Y}/E_{R})$$

(23) $$S_{Y}=S\cdot f/(f_{Y}+f_{R}\cdot E_{R}/E_{Y})$$

In the case where the failure of a component working in the plastic range has occurred, the value l_Y_ = (1 + *ε*_Y_) is substituted in place of the initial length in the plastic part l_Y_ = 1. Then, in both parts of the specimen, with zero external loads, the maximum stress is determined from the system of equations:

(24) $$\left\{\begin{array}{l} \Delta S_{R}\cdot f_{R}=\Delta S_{Y}\cdot f_{Y},\\ 1+\frac{\Delta S_{R}}{E_{R}}=(1+\varepsilon_{Y})\left(1-\frac{\Delta S_{Y}}{E_{Y}}\right). \end{array}\right.$$

Solving the system of equations in the elastic range allows it to be written in the form:

(25) $$\Delta S_{R}=E_{R}\cdot \varepsilon_{Y}/\left(1+(1+\varepsilon_{Y})f_{R}E_{R}/f_{Y}E_{Y}\right)$$

(26) $$\Delta S_{Y}=E_{Y}\cdot \varepsilon_{Y}/\left(1+\varepsilon_{Y}+f_{Y}E_{Y}/f_{R}E_{R}\right)$$

Assuming that the elongation of the working part in the plastic range is proportional to the number of plastic failures (parts that have reached their yield point), we obtain:

$$\varepsilon_{Y}=\varepsilon_{Y1}\cdot (i_{Y}-1),\;i_{Y}=1,\ldots ,r_{Y},$$

where *ε_Y_*_1_ is a parameter of the model.

(27) $$\left\{p_{ij}^{\left(k\right)}\right\}=P^{k}=\left[\begin{array}{cc} I & O\\ R^{\prime } & Q^{k} \end{array}\right]$$

where

Q is a stochastic matrix describing the probability of transformation only within transients;
I is a unity matrix;
0 is a matrix containing zeros (r-s) through s;
R is a matrix describing the probability of transformation from transition states to absorbing states within a single step.

The $Q^{k}$ matrix components (i, j) are related to the probability of reaching the transition state S_j_ after precisely k steps from the (transition) state S_i_.

### 4.4. Determination of Residual Strength

Based on the assumption that one step in the Markov chain related to k_M_ cycles (where k_M_ is also an element of the vector η) in the cyclic load, the column vector of the average number of steps before the transformation (of different starting states—transitions) can be calculated with Equation (27). The variance vector (28) in the probability matrix (29) in the absorbing condition (namely, the components of the first row of Matrix B) show the probability of different types of destruction (of elements operating in the elastic range with unacceptable elongation of the samples within the plastic range, or under conditions of combination of these destructive factors).

τ_2_=(2N-I)τ-τ_sq_, (28)

where

$\tau_{sg}\;(i)=(\tau (i){)}^{2},i\in I_{A}$, and *I_A_* is a sequence of indices of irreversible states.

(29) $$B=\{B_{ij}\;\}=NR,$$

where

*B_ij_* is the probability in the absorbing state of the process at the *j*-th transformation state if the initial state is the *i*-th irreversible state.

The fatigue strength *t_p_(S)* impacts a number of cycles via the relation 19, (namely, the probability *p* of destruction at the initial normal stress S—the fatigue curve)

(30) $$t_{p}(S)=k_{M}F_{T_{A}}^{-1}\;(p;S,\eta )$$

and the probability vector after n_1_ steps with stress S_1_ is defined as (only for undamaged samples):

(31) $$\pi_{S_{1}n_{1}}=(1,0,\ldots )P_{1}^{n_{1}}$$

The components of the probability distribution vector of the unabsorbed (irreversible) states of the Markov chain take the following form:

(32) $$\pi_{S_{1}n_{1}}^{*}\left(k\right)=\pi_{S_{1}n_{1}}(k)/\sum_{m=1}^{m^{*}}\pi_{S_{1}n_{1}}(m)$$

where ${\;\pi }_{S_{1}n_{1}}^{*}(k)$, *k* = 1, …, m^*^ are vector components $\pi_{S_{1}n_{1}}$;

m^*^= (r_Y_ + 1)(r_R_ + 1) − (r_Y_ + 1 + r_R_) is the total number of unabsorbed (irreversible) states.

The last (r_Y_ + 1 + r_R_) components of the vector π_n1*_, related to the transformation states, are 0, because only the samples that were not destroyed after the initial loading were taken into consideration. For such samples, the stress distribution function σ_n1_^II^, at which the transition takes place in a single step of the Markov chain (which corresponds to the destruction of the sample in k_M_ cycles), equals

(33) $$F_{\sigma_{n1}}(x)=\pi_{S_{1}n_{1}}^{*}P(x)b,\;$$

where x ≥ S_1_, *P(x)* is a probability transformation matrix with *S = x*.

**Figure 4:**
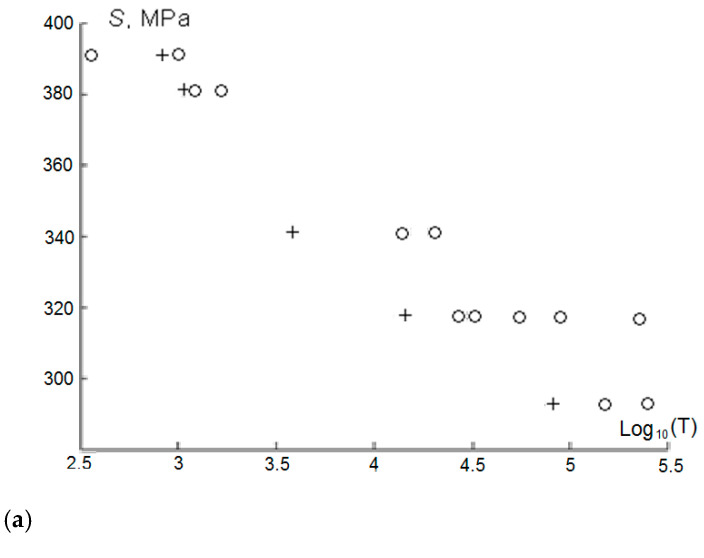

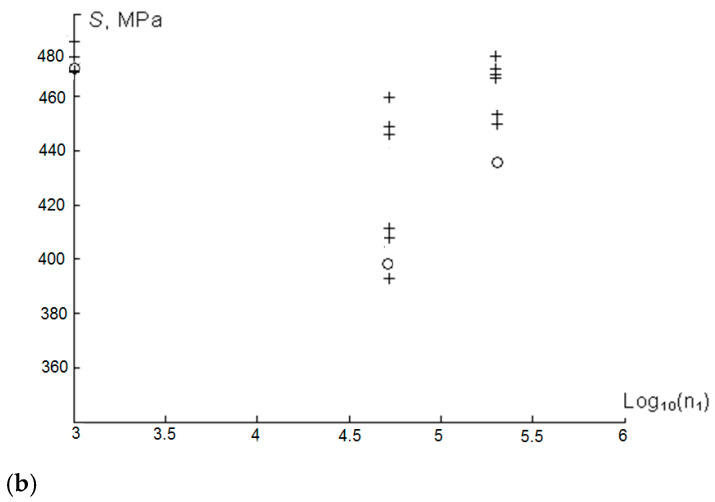
Estimation of the average fatigue strength (**a**) and residual strength (**b**) of the epoxy joint before (+) and after thermal shocks (o) for loads *(S_1_, n_1_)* (Table 3 and Table 4).

Estimation of the mean fatigue *E(T(S))* at any *S* (for our example, *S* = 57.0 MPa and *S*= 44.6 MPa as the failure stresses of single-layer epoxy joints after thermal shocks relative to the specimens before thermal shocks) with the specified parameters of the model (Table 9) consequently gives a fairly accurate representation of the fatigue curve, i.e., matching (Figure 4) the fatigue curve data (T-N) to the experimental results from the number of initial loads with a starting frequency of 5 Hz at two stress levels K * *S_statist_* (K_0.1_ = 0.3; 0.4).

With that aim, a number of cyclic loads with quite big values were assumed to approximately equal the minimal significance of fatigue strength at a specific load level determined on the basis of the calculations.

## 5. Conclusions

This paper describes a model for determining the strength of a fibre composite based on critical micro-volumes, taking into account the failure stresses of single-layer epoxy joints after thermal shocks relative to the samples before thermal shocks. The non-linear internal source of heat and heat transfer result in heterogeneity in the temperature and degree of curing within the epoxy part. The use of the Markov model allowed for a probabilistic analysis of material degradation under the assumed cyclic loading conditions, as well as analysis of the influence of parameter uncertainty on the predicted residual life. The analysis enabled us to consider the variables related to the experimentally based cure, namely the resin system, which can improve the mechanical and performance properties of the finished part by reducing the deformation and residual stresses during the cure process.

## Figures and Tables

**Table 1:** Preparation technologies for specimens made from the adhesive composition (LH-160 with the addition of hardener H-147 at 25% [29]).

Designation of Sample Series	P1	P2	P3	P4
Sample type	- Against thermal shocks, - Against reheating	- After thermal shocks (500 cycles: +60°C/−40°C), - Against reheating	- Against thermal shocks with reheating (80°C for 2 h)	- After thermal shocks (500 cycles: +60°C/−40°C) with reheating (80°C for 2 h)

**Table 2:** Statistical analysis [43] of the static strength (*S*) and Young’s modulus (E) of fibre bundles and epoxy–glass composite specimens with different L_BP_ (explanations under the table).

Mechanical Properties of the Composite Components with Different L_BP_	Type Criterion OSPPt	Calfa	Average *S_stati._*	Standard Deviation	N_specimens_
**Log-normal distribution *S_stat._*, MPa**					
*S*_EPOXY_C1_	0.27439	0.31162	6.590	0.14987	20
*S*_EPOXY_C2_	0.18322	0.26505	6.544	0.13653	31
*S*_EPOXY_C3_	0.24046	0.34601	6.411	0.076333	15
*S*_EPOXY_A_	0.21924	0.29487	6.076	0.065701	23
*S*_EPOXY_B_	0.14194	0.30321	6.189	0.059936	21
**Normal distribution *S_stat._*, MPa**					
*S*_EPOXY_C1_	0.33237	0.33977	573.017	89.3891	20
*S*_EPOXY_C2_	0.23027	0.26217	621.303	86.9879	31
*S*_EPOXY_C3_	0.25157	0.34707	610.233	46.797	15
*S*_EPOXY_A_	0.20613	0.30016	436.071	28.3748	23
*S*_EPOXY_B_	0.1553	0.30878	487.561	29.4538	21
**Mechanical Properties of the Composite’s Components** **with Different L_BP_ Values**	**Type criterion OSPPt**	**Calfa**	**AverageE**	**Standard Deviation**	**N_specimens_**
**Log-normal distribution E, GPa**					
E_EPOXY_C1_	0.30161	0.31136	2.681	0.051224	20
E_EPOXY_C2_	0.34004	0.26276	3.337	0.032427	31
E_EPOXY_C3_	0.36118	0.34924	3.328	0.025142	15
E_EPOXY_A_	0.21243	0.29428	3.119	0.050672	23
E_EPOXY_B_	0.23458	0.30164	3.085	0.072699	21
**Normal distribution E, GPa**					
E_EPOXY_C1_	0.27588	0.31267	17.854	0.89356	20
E_EPOXY_C2_	0.32463	0.26123	28.139	0.89524	31
E_EPOXY_C3_	0.37838	0.34558	27.906	0.71101	15
E_EPOXY_A_	0.20497	0.29609	22.645	1.1382	23
E_EPOXY_B_	0.22871	0.30602	21.929	1.5821	21

Indications: *S*, *E*_EPOXYA, EPOXY_C2_—strength and Young’s modulus of the specimens (EPOXY_A) and the elementary fabric component beam with L_BP_ = 250 mm. *S*, *E*_EPOXY_B, EPOXY_C1_—strength and Young’s modulus of the specimens (EPOXY_B) and the elementary fabric component beam (EPOXY_C1) with L_BP_ = 120 mm. *S*, *E*_EPOXY_C3_—strength and Young’s modulus of the elementary fabric component beam (EPOXY_C3) with L_BP_ = 450 mm.

**Table 3:** Statistical treatment parameters of the average strength of epoxy matrix composite samples with different L_BP_ values.

Specimens *S_stat._* with Different L_BP_ Values	D*	Average, *S_stat_.,* MPa	Dispersion	Standard Deviation	The Criterion S-K [43]
SHEPOXY_A	**0.113879**	6.0758	0.003421	0.065701	**0.516735** < 0.99
SHEPOXY_B	**0.135569**	6.1891	0.004986	0.059936	**0.60825** < 0.99

**Table 4:** Fatigue parameters of the composite with the structure [0/45/0] at R = 0.1.

S_HEPOXY_B (*R* = 0,1)_, MPa	*N*, Cycles
292.535	147.000; 241.000
317.36	28.500; 31.000; 36.800; 56.000; 92.000; 222.000
341.29	13.700; 14.600; 19.650
380.78	300; 450; 1100; 1200; 1650; 1700
390.05	350; 1000

**Table 5:** Residual strength values for the composite [0/45/0] and R = 0.1.

S_HEPOXY_A (*R* = 0,1)_, MPa	*N*, Cycles	*S_R_*, MPa	Average *S_R_*, MPa
243.78	265.000	399.8	399.8
292.53	60.000	465.84; 432.04; 425.13; 414.73; 408.84; 387.55	422
390.05	900	481.58; 478.39; 477.78; 474.16; 456.54; 451.85	470

**Table 6:** Exemplary structure of the probability transformation matrix [38].

		$j_{y}$	**1**			**2**			**3**		
		$j_{R}$	**1**	**2**	**3**	**1**	**2**	**3**	**1**	**2**	**3**
$i_{y}$	$i_{R}$	i\j	**1**	**2**	**3**	**4**	**5**	**6**	**7**	**8**	**9**
**1**	**1**	**1**	$p_{R0}p_{Y0}$	$p_{R1}p_{Y0}$	$p_{R2}p_{Y0}$	$p_{R0}p_{Y1}$	$p_{R1}p_{Y1}$	$p_{R2}p_{Y1}$	$p_{R0}p_{Y2}$	$p_{R1}p_{Y2}$	$p_{R2}p_{Y2}$
	**2**	**2**	0	0	$p_{R1}p_{Y0}$	0	$p_{R0}p_{Y1}$	$p_{R1}p_{Y1}$	0	$p_{R0}p_{Y2}$	$p_{R1}p_{Y2}$
	**3**	**3**	0	0	1	0	0	0	0	0	0
**2**	**1**	**4**	0	0	0	$p_{R0}p_{Y0}$	$p_{R1}p_{Y0}$	$p_{R2}p_{Y0}$	$p_{R0}p_{Y1}$	$p_{R1}p_{Y1}$	$p_{R2}p_{Y1}$
	**2**	**5**	0	0	0	0	$p_{R0}p_{Y0}$	$p_{R1}p_{Y0}$	0	$p_{R1}p_{Y0}$	$p_{R1}p_{Y1}$
	**3**	**6**	0	0	0	0	0	1	0	0	0
**3**	**1**	**7**	0	0	0	0	0	0	1	0	0
	**2**	**8**	0	0	0	0	0	0	0	1	0
	**3**	**9**	0	0	0	0	0	0	0	0	1

**Table 7:** Parameters of the model [37].

Characteristics	Dependencies
Fatigue strength (time to absorption)	*T = X_1_ + X_2_ +...+ X_r_* (8) where: *X_i_*, *i* = 1; *r*—destruction time (is) in an i-m state
Random variable X_i_ in a geometric distribution	*P*(*X_i_ = n*)* = *(1* − p*)*^n−*1*^p_i_* (9)
Expected value	*E(X_i_) = 1/p_i_* (10)
Dispersion	V(X_i_) = (1 − p_i_)/p_i_^2^ (11)
Random variable	ET=∑i=1r1pi (12) $V\left(T\right)=\sum_{i=1}^{r}\frac{\left(1-p_{i}\right)}{p_{i}^{2}}$ (13)
Function which produces the probabilities of the random variable T	$G_{T}\left(z\right)=\sum_{i=0}^{\infty }p_{T}(i)\cdot z^{i}\prod_{i=1}^{\infty }\frac{zp_{i}}{1-z(1-p_{i})}$
Function of cumulative distribution	*F_T_*(*t*)* = p_*1* r*+1*_*(*t*)**, t = 1, 2, 3 (14) where: *p_*1* r*+1*_*(*t*)** is (1, *r* +1)—matrix element
Function of fatigue strength distribution	*P*(*t*)* = P^t^* described as: *F_T_*(*t*)* = a P^t^ b* (15) where: *a* = (100....); b = (0.0,…0.1)T- column vector

**Table 8:** Model conditions [36,37].

Model Conditions
$1.N=\{E(T_{ij})\}={\left(1-Q\right)}^{-1}$ basic matrix of the different initial states. $2.\tau =\{E(T_{ij})\}=N\xi ,$ where ξ = [1,…,1] is a columnar unit vector. $3.\tau_{2}=(2N-I)\tau -\tau_{sg}$ $\mathrm{where}:\tau_{sq}=\left\{{\left(E\left(T_{i}\right)\right)}^{2}\right\}.$ 4.B=NR probability matrix in the absorbing state. where: *T_ij_* is the number of visits to state *j*, starting from state *i*; *T_i_* is the time of absorption (considering also the initial state) starting from state *i*; *E(T_i_), Var(T_i_)* are the average and variance of the absorption time if i is the initial state; *τ = {E(T_i_}), τ_2_ = {Var(T_i_)}* are the corresponding column vectors, i is the transition state index; *B = {b_ij_}* is the probability matrix of absorption; *b_ij_* is the probability that the process will be absorbed in state j if the initial state is i.

**Table 9:** Model parameters.

Model Parameters	Parameter Values
Number of working elements in the critical micro-volume, *r_Y_*	5
Average strength value for longitudinal components operating in the elastic range, Q_OR_(exp(Q_OR_))	6.1883 (487.56 MPa) *
Standard deviation of the element’s longitudinal strength, $Q_{1R}$	0.15
Standard deviation in the critical plastic part of micro-volumes, $Q_{1Y}$	0.2
Number of cycles equivalent to a single step in the Markov chain, *k_M_*	227

* Brackets contain values in the linear scale.

## Data Availability

The original contributions presented in this study are included in the article. Further inquiries can be directed to the corresponding author.
